# Supplementary material for: Caspase-6 Is a Non-Apoptotic Effector of Shear-Induced Morphological Adaptation in Pulmonary Artery Endothelial Cells In Vitro
Source: Cells. 2025 Oct 25;14(21):1669. doi: 10.3390/cells14211669 (PMC12609614; doi:10.3390/cells14211669)
Supplement: Supplementary file 1 [file cells-14-01669-s001.zip › cells-3874402-supplementary.pdf]

Supplementary Figures:

Caspase-6 Is a Non-Apoptotic Effector of Shear-Induced Morphological Adaptation in Pulmonary Artery Endothelial Cells In Vitro

Corey Wittig, Emir Bora Akmeriç, Laura Michalick, Jakob M. König, Wolfgang M. Kuebler, Holger Gerhardt, and Robert Szulcek

This file includes:

- Figures S1 and S2

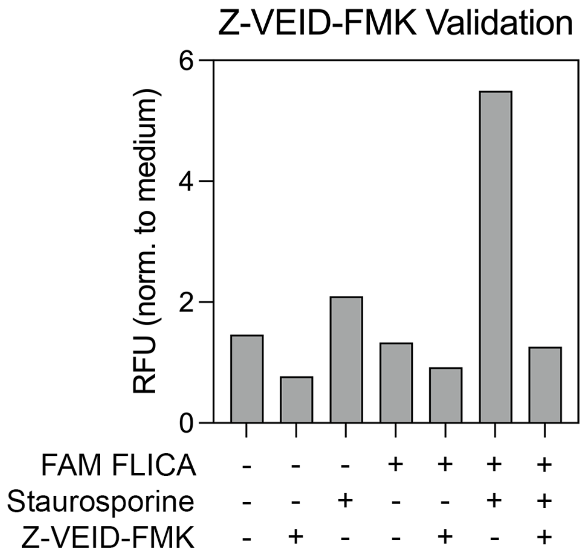

Figure S1. Validation of caspase-6 inhibition using Z-VEID-FMK in hPAEC. Human pulmonary artery endothelial cells (hPAEC) were treated in triplicate with 200 nM staurosporine for 4 hours at 37 °C to induce caspase activation, with or without 2 μM Z-VEID-FMK caspase-6 inhibitors maintained during treatment. Caspase-6 activity was measured using FAM FLICA fluorescent caspase-6 probes, and endpoint fluorescence was measured using a fluorescence plate reader. Relative fluorescence units (RFU) were normalized to cell-free medium controls. The relative fluorescence units (RFU) of the staurosporine-stimulated hPAEC without Z-VEID-FMK caspase-6 inhibitors was 4.4-fold higher than with Z-VEID-FMK caspase-6 inhibitors (5.50 vs. 1.26 RFU, respectively), which produced RFU values comparable to unstimulated hPAEC (1.26 vs. 1.33 RFU, respectively), indicating the Z-VEID-FMK caspase-6 inhibitor fully inhibited caspase-6 activity.

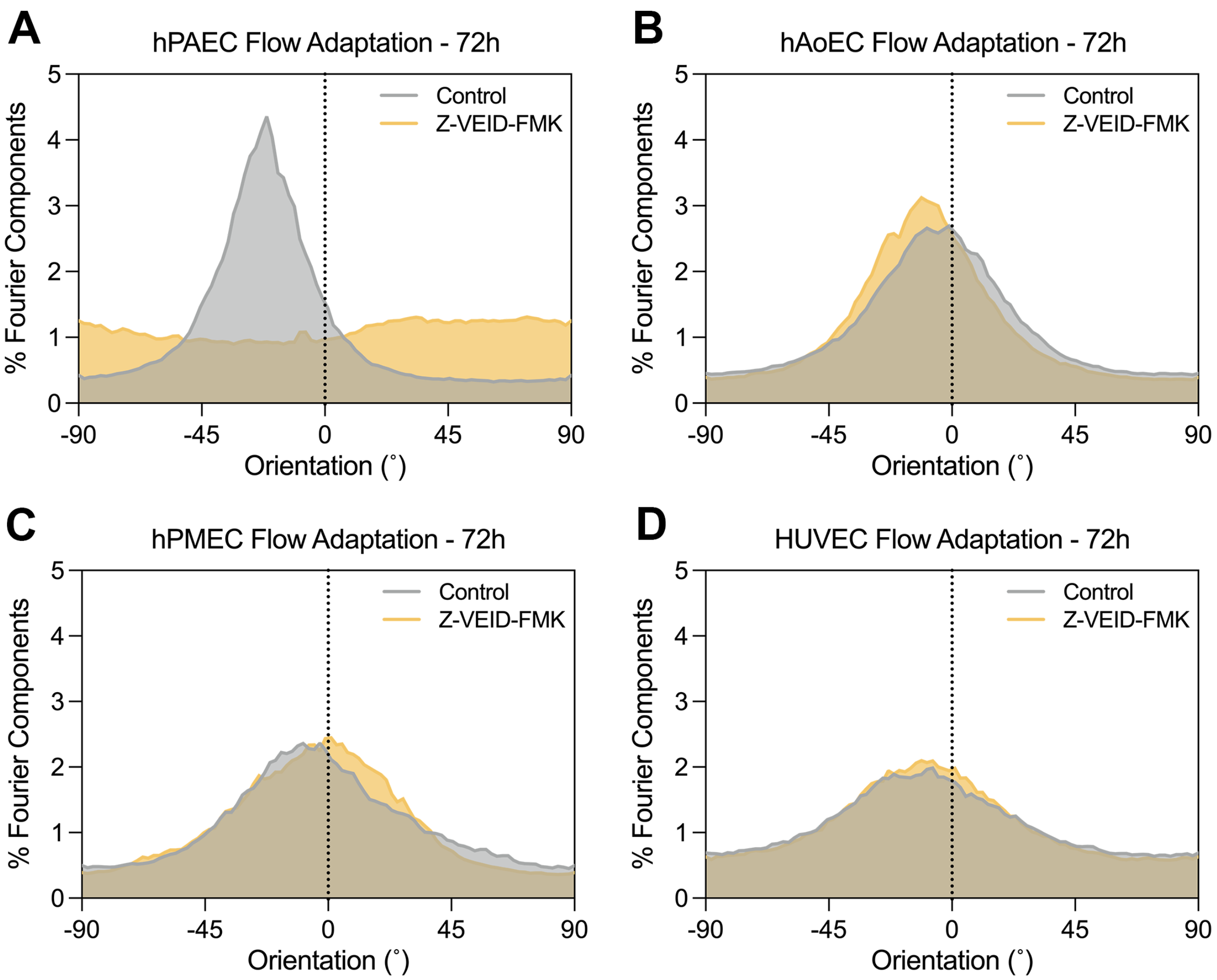

Figure S2. Shear adaptation in caspase-6-inhibited endothelial cells from different vascular beds. **(A)** Human pulmonary artery endothelial cells (hPAEC, n = 3 pooled), **(B)** human aortic endothelial cells (hAoEC, n = 1), **(C)** human pulmonary microvascular endothelial cells (hPMEC, n = 1), and **(D)** human umbilical vein endothelial cells (HUVEC, n = 1) were subjected to 15 dyn/cm^2 unidirectional, laminar fluid flow for 72 hours with or without constant caspase-6 inhibition using 2 μM Z-VEID-FMK caspase-6 inhibitors in vitro. Adaptation parallel to the direction of fluid flow (0°) was quantified using the Directionality 2.3.0 ImageJ plugin. Only hPAEC showed an effect due to Z-VEID-FMK administration, with a complete suppression of shear adaptation. Five images at 10× magnification were used per condition.
